# RETRACTED: Yuan et al. Green Remanufacturer’s Mixed Collection Channel Strategy Considering Enterprise’s Environmental Responsibility and the Fairness Concern in Reverse Green Supply Chain. *Int. J. Environ. Res. Public Health* **2021**, *18*, 3405

**DOI:** 10.3390/ijerph22081254

**Published:** 2025-08-11

**Authors:** Xigang Yuan, Fei Tang, Dalin Zhang, Xiaoqing Zhang

**Affiliations:** 1 Business School, Jiangsu Normal University, Xuzhou 221116, China; yxg200811606@126.com (X.Y.); xqzhang22@163.com (X.Z.); 2 School of Economics and Management, Southwest Jiaotong University, Chengdu 610031, China; tangf88@my.swjtu.edu.cn; 3 Department of Computer Science, Aalborg University, 9220 Aalborg Øst, Denmark

The journal retracts the article “Green Remanufacturer’s Mixed Collection Channel Strategy Considering Enterprise’s Environmental Responsibility and the Fairness Concern in Reverse Green Supply Chain” [1] cited above.

Following publication, the authors contacted the Editorial Office to raise concerns relating to major scientific errors in the theoretical model contained within this study [1].

Adhering to our standard procedure, the Editorial Board evaluated and confirmed flaws in the presented theoretical model. As a result, the Editorial Board and the authors agreed that these errors compromise the validity of the overall findings and mutually decided to retract this publication [1], as per MDPI’s retraction policy (https://www.mdpi.com/ethics#_bookmark30).

This retraction was approved by the Editor-in-Chief of the journal *IJERPH*.

The authors agreed to this retraction.
